# The benefits of edible mushroom polysaccharides for health and their influence on gut microbiota: a review

**DOI:** 10.3389/fnut.2023.1213010

**Published:** 2023-07-06

**Authors:** Qilong Zhao, Yu Jiang, Qian Zhao, Habasi Patrick Manzi, Li Su, Diru Liu, Xiaodan Huang, Danfeng Long, Zhenchuang Tang, Ying Zhang

**Affiliations:** ^1^School of Public Health, Lanzhou University, Lanzhou, China; ^2^Institute of Food and Nutrition Development, Ministry of Agriculture and Rural Affairs, Beijing, China

**Keywords:** edible mushroom polysaccharides, gut microbiota, host health, short chain fatty acids, beneficial effects

## Abstract

The gut microbiome is a complex biological community that deeply affects various aspects of human health, including dietary intake, disease progression, drug metabolism, and immune system regulation. Edible mushroom polysaccharides (EMPs) are bioactive fibers derived from mushrooms that possess a range of beneficial properties, including anti-tumor, antioxidant, antiviral, hypoglycemic, and immunomodulatory effects. Studies have demonstrated that EMPs are resistant to human digestive enzymes and serve as a crucial source of energy for the gut microbiome, promoting the growth of beneficial bacteria. EMPs also positively impact human health by modulating the composition of the gut microbiome. This review discusses the extraction and purification processes of EMPs, their potential to improve health conditions by regulating the composition of the gut microbiome, and their application prospects. Furthermore, this paper provides valuable guidance and recommendations for future studies on EMPs consumption in disease management.

## 1. Introduction

**Figure 1:**
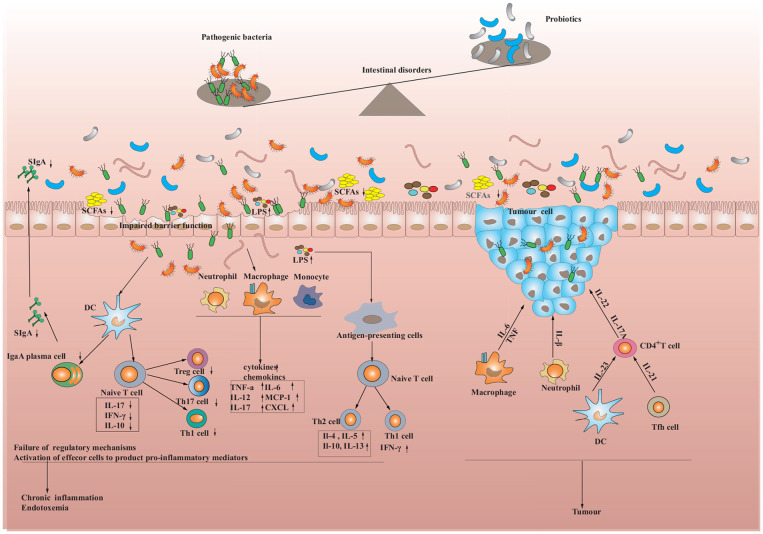
Intestinal dysbiosis and the immune response in the intestine. When pathogens are encountered, dendritic cells capture the pathogens' antigens through epithelial cells, which are then processed. The processed antigens are presented to T cells and trigger their activation. Activated T cells stimulate the secretion of cytokines by epithelial cells, which in turn increases the production of antimicrobial peptides. Meanwhile, T cells and dendritic cells can also activate B cells, transforming them into plasma cells that produce Immunoglobulin A (IgA), which as a consequence provides further resistance against pathogen invasion. However, if the immune cells continue to activate or the pathogens persist in stimulating the immune system, the functioning of regulatory cells may be suppressed, leading to chronic inflammation and the development of metabolic diseases, inflammatory bowel diseases (IBD), and tumors.

The gut microenvironment consists of physicochemical conditions and the gut microbiome, which are interdependent and both are critical to body function. Its essential role in supporting overall health stems from its involvement in metabolic regulation, signaling pathways, synthesis of crucial nutrients, and maturation of immune cells, among other functions (1, 2). However, dysbiosis of the gut microbiota can result from external factors such as alterations in the environment, diet, or the use of antibiotics and other medications. This condition, also referred to as intestinal ecological dysbiosis or gut dysbiosis, disrupts the normal balance of microorganisms in the gut, leading to a loss of immune balance and the development of diseases. The three main types of gut microbiota dysbiosis are disproportionate, translocation, and autoinfection. Typically, the gut microbiota and the host are interdependent and mutually regulated, establishing a dynamic equilibrium. However, any changes to the internal or external environment can disrupt this balance, leading to dysbiosis of the gut flora, as depicted in Figure 1. Multiple studies have demonstrated that disruptions in the intestinal ecosystem are linked to various gastrointestinal and extraintestinal metabolic disorders, including but not limited to obesity, type 2 diabetes, cardiovascular disease, and cancer (3–6). Among external factors, diet plays a significant role in inducing short-term shifts in the gut microbiome. Both digestible and non-digestible carbohydrates, proteins, lipids, polyphenols, probiotics, and prebiotics can affect gut ecology. Therefore, it is evident that diet can exert control over the gut microbiota to improve overall health (7, 8).

Mushrooms, which are macroscopic fruiting bodies produced by basidiomycete and ascomycete fungi, have gained a diverse range of reputations throughout history. Ancient Egyptians regarded them as “sons of the gods”, while ancient Chinese believed they were the “elixir of life” (9, 10). Currently, various species of mushrooms are commonly consumed, including for example *Grifola frondosa, Hericium erinaceus, Flammulina velutipes, Auricularia auricula, Lentinula edodes*, and *Ganoderma lucidum*. Around the world at least 2,786 mushroom species are consumed in 99 countries (11). Mushrooms are a rich source of essential nutrients such as proteins, carbohydrates, fats, dietary fiber, and vitamins (12). In addition to their nutritional value, various bioactive compounds can be found in mushrooms, including β-glucans, polysaccharide-protein complexes, lectins, polysaccharide-peptides, terpenoids, sterols, alkaloids, and phenolic compounds (13–15). Polysaccharides, in particular, have garnered significant attention due to their notable physiological effects, including immunomodulatory, antioxidant, antitumor, antiviral, anti-carcinogenic, and anti-inflammatory activities (16, 17). Currently, the interaction between edible mushroom polysaccharides (EMPs) and the intestinal microbiota, as well as the development of related dietary interventions, are heavily researched topics in the international arena. As such, this review offers a thorough exploration of the extraction and purification of EMPs, their potential for regulating intestinal microbiota and promoting health, and their promising prospects for applications.

## 2. EMPs: extraction, purification, and characterization

**Figure 2:**
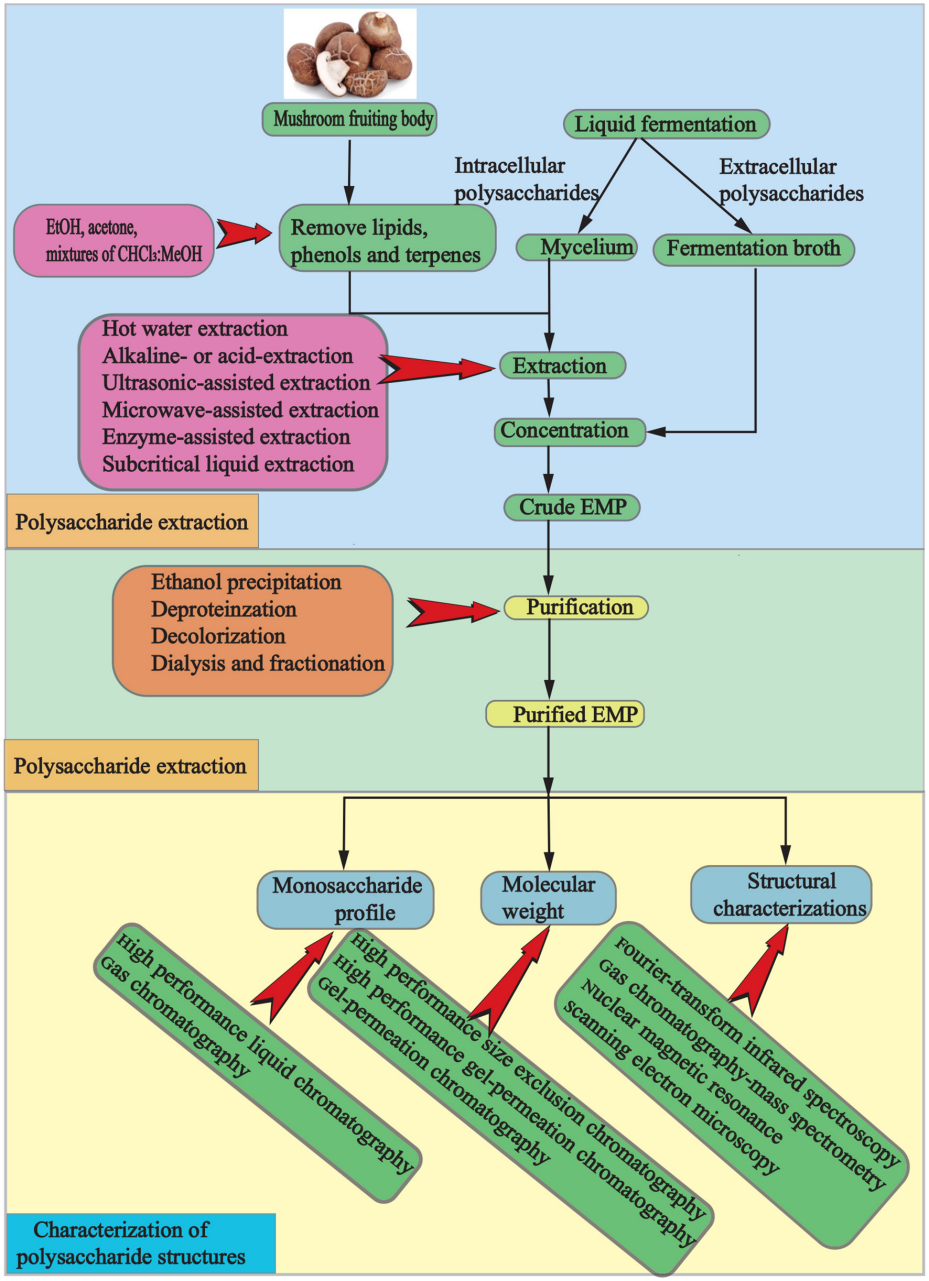
A schematic representation of the process for extracting, purifying, and characterizing polysaccharides from mushrooms.

EMPs, which are polar macromolecular compounds, can be obtained through a process involving isolation, extraction, and purification. Initially, the dried mushroom is ground, and terpenes, phenols, and lipids are removed using alcohol or organic solvents. Crude EMPs are then extracted with hot water, and ethanol precipitation, deproteinization, dialysis, and fractionation are used for purification (Figure 2). However, traditional processes are hindered by high costs, environmental pollution, potential degradation and condensation of active ingredients, and time consumption. To overcome these limitations, advanced techniques such as microwave-assisted extraction, enzymatic-assisted extraction, ultrasound-assisted extraction, high pressure-assisted extraction, pulsed electric-assisted extraction, and pressurized hot water extraction have been developed (2). These techniques differ in several aspects (Tables 1, 2).

**Table 1:** Comparison between different mushroom polysaccharides extraction techniques (18, 19).

**Technique**	**Advantages**	**Shortcomings**	**Factors of influencing yields**
Hot water extraction	Simple equipment requirements and low operation coast	Time-comsuption, high operating temperature, excess energy requirement, solvents waste	Treatment temperature, extraction time
Alkaline- or acid-extraction	Selectively extraction	High operating temperatures, time-comsuption up to 24 h, side-products, compounds degradation	Treatment temperature, extraction time
Ultrasonic-assisted extraction	Lower solvent consumption, low energy consumption, high processing throughput, shorter processing time, simple operation	Difficult to monitor the temperature for low repeatability	Ultrasonic power, liquid-solid ratio, frequency, pulse duration and interval, extraction temperature and time
Microwave-assisted extraction	Lower solvent consumption, flexible, less processing time, high extraction yield,	Inhomogeneous heating, destroy chemical structure	Microwave power, treatment temperature, extraction time
Enzyme-assisted extraction	Easy operation, high specificity and efficiency, low energy requirements, operating temperature, environmental-friendly	Relatively high cost of enzymes and difficulty to purify	The type and concentration of enzyme, reaction time, temperature, the liquid-solid ratio, pH value
Subcritical liquid extraction	Economical and environment-friendly, improved extraction speed and yield, good reproducibility	Higher cost	Temperature, pressure, the liquid-solid ratio, extraction time and frequency

**Table 2:** Extraction effect of different extraction methods.

**Source of EMPs**	**Extraction method**	**Extraction condition**	**Yield**	**References**
*Volvariella volvacea*	Hot water	100°C for 3 h.	15.58%	(20)
*Volvariella volvacea*	Microwave-assisted	600 W for 30 min.	11.05%	(20)
*Volvariella volvacea*	Ultrasonic-assisted	40 kHz for 30 min.	9.06%	(20)
*Pleurotus ostreatus*	Subcritical water	Temperatures 180°C.	20.35%	(21)
*Rugiboletus extremiorientalis*	Hot water	Temperature (75°C), precipitation with ethanol (3:1 v/v), and time (3 h).	9.67%	(22)
*Morchella esculenta*	Hot water	Temperature (80°C), precipitation with ethanol (3:1 v/v), and time (2 h).	3%	(23)
*Coriolus versicolor*	Hot water	Water at 100°C for 8 h, and then precipitated with ethanol at 4°C for 13 h.	5.6%	(24)
*Pleurotus eryngii*	Cold water	Cold-water extraction at room temperature for 6 h.	1.2%	(25)
*Pleurotus eryngii*	Hot water	Hot-water extraction at 100°C for 6 h.	7.0%	(25)
*Pleurotus eryngii*	Autoclave	autoclave extraction at 121°C for 30 min.	1.3%	(25)
*Hypsizygus ulmarius*	Hot water	Temperature (100°C), time (3 h), pH (7.0) and ratio of material to solvent (1:25).	12.53%	(26)
*Morchella sextelata*	Subcritical water	Temperature (150°C), pressure (5 MPa), time (30 min), and ratio of material to solvent (1:20).	MSP1-1(43.3%), MSP1-2(18.2%)	(27)
*Polyporus umbellatus*	Hot water and graded alcohol precipitation	Temperature (100°C), time (2 h), precipitation with ethanol (30, 60, and 80%).	3.87, 5.82, and 5.38%	(28)
*G. lemaneiformis*	Ultrasound-microwave-assisted	Temperature (87°C), 50 W, 31.7 min,1.0:60.7 (w/v).	34.84%	(18)
*Lentinus edodes*	Subcritical water	Temperature (100, 110, 120, 130, and 140°C), time (15 min).	11.08, 13.27, 14.11, 12.57, and 12.65%	(29)
*Morchella esculenta*	Pulsed electric field assisted	Electric field strength (19 ± 1 kV/cm), pulse number (6 ± 1), solid-liquid ratio of (28.5 ± 1.5 mL/g).	56 μg/mL	(30)
*Cordyceps cicadae*	Two-phase aqueous	Temperature (61°C), ammonium sulfate concentration (18%), ethanol concentration (40%), feed-to-liquid ratio (33 mL/g), time (60 min).	6.96 ± 0.11%	(31)

The extraction method and purification parameters can significantly influence the interpretation of biological activity of polysaccharides (18). Hence, it is crucial to carefully consider these factors, such as volume, solvent type, pH, time, and temperature, when extracting polysaccharides to preserve their biological activity and achieve the desired extraction yield. Additionally, combining different extraction procedures may also help to improve recovery and maintain the bioactivity of EMPs. Kinetic models and mathematical tools can also be utilized to forecast the yield of EMPs under specific conditions (32). For example, In a study on *Agrocybe aegerita* polysaccharides, the response surface methodology (RSM) was used to optimize the accelerated solvent extraction parameters, and the results showed that the optimal conditions were water as extraction solvent, extraction temperature of 71°C, extraction time of 6.5 min, number of cycles of 3, extraction pressure of 10 MPa, and extraction yield of 19.77% (33).

Typically, raw EMPs often contain various “contaminants,” including proteins, inorganic salts, lignin, amino acids, and other substances (34). Therefore, a purification process is necessary to obtain pure EMPs. One common approach for removing proteins from raw EMPs is to use the Sevag method, trichloroacetic acid method, or enzymatic hydrolysis method (35). However, the Sevag method can be time-consuming and complicated, so a combination of enzymatic hydrolysis and Sevag methods is often used to minimize the loss of polysaccharides. After protein removal, activated carbon, macroporous resin, or hydrogen peroxide can be used to decolorize the raw polysaccharide extracts. However, as the extracts are rich in negative ions such as ketones, phenols, and quinones, decolorization using the activated carbon method may not be effective. In contrast, the hydrogen peroxide method is effective in decolorizing negative ions (36). Recently, adsorption resins or ion exchange resins have become popular for decolorization due to their stable characteristic group structure and high decolorization rate (34). To analyze the molecular weight, monosaccharide composition, and glycosidic bond configuration of polysaccharides, further separation and purification of raw EMPs through ion exchange chromatography and gel chromatography is necessary to obtain homogeneous polysaccharides based on the purification process described above (37).

EMPs are categorized into primary and advanced structures (34, 38, 39). The primary structure focuses on the components of the main chain and branching chains, whereas the advanced structure encompasses the conformation between the main chains and non-covalent bonding between EMPs. The chemical characterization of polysaccharides is critical, as their activity is closely tied to their structure. Currently, structural characterization of EMPs mainly involves analysis of their primary structure, such as total sugar content, molecular weight size and distribution, monosaccharide composition and molar ratio, and glycosidic bond type (40–43). Methods for determining EMPs content include the anthrone-sulfuric acid method, phenol-sulfuric acid method, colorimetric method, high-performance liquid chromatography (HPLC) method, enzymatic method, DNS reduction method, and ion exchange chromatography (44). Research has indicated that immune cells display distinct affinities toward high molecular weights, resulting in varied therapeutic outcomes (45). Hence, exploring the size of molecular weight in polysaccharides derived from mushrooms is imperative. High-performance liquid chromatography (HPLC), high-performance gel filtration chromatography (HPGFC), high-performance gel chromatography (HPGPC), polyacrylamide gel electrophoresis, and ultrafiltration retention method are used to determine molecular weight (18, 46, 47), while gas chromatography (GC), high-performance liquid chromatography (HPLC), and capillary electrophoresis are applied to identify monosaccharide composition (48). The advanced structure encompasses supramolecular structure, solution conformation, reticular structure, and is analyzed through X-ray diffraction, fluorescence correlation spectroscopy, molecular modeling, and atomic force microscopy (49, 50). The structure of hydrolyzed and derivatized EMPs can be characterized through high-performance liquid chromatography (HPLC), infrared spectroscopy (IR), gas chromatography-mass spectrometry (GC-MS), 1 hydrogen-nuclear magnetic resonance (1H-NMR), and atomic force microscopy (AFM) (18, 51). EMPs sugar residue amount and composition, glycosidic bond type, anomeric carbon configuration, attachment sites, and sequence can be expressed through 1D and 2D nuclear magnetic resonance (NMR) spectroscopy (52, 53). At this point, there are limited studies on the structural characterization of EMPs. However, the determination of polysaccharide structure remains an ongoing area of exploration. The methods currently available are not fully developed and have several drawbacks, such as the high molecular weight of polysaccharides, difficulties in UV absorption, and limited control over qualitative and quantitative analysis after purification. These limitations hinder the advancement and utilization of EMPs to a certain extent.

## 3. Regulatory effects of EMPs on intestinal bacteria

### 3.1. Enzymes from intestinal bacteria hydrolyze EMPs

The degradation of EMPs in the colon is facilitated by a group of enzymes known as carbohydrate-active enzymes (CAZymes) (54). While the human genome encodes only 8–17 GH enzymes with limited capability to digest complex carbohydrates, more than 10,000 CAZymes that can break down complex carbohydrates have been identified in 177 reference bacterial genomes (55). This indicates that the gut microbiota is responsible for the degradation of various polysaccharides, including β-glucans, into monosaccharides or oligosaccharides, which can modulate the intestinal microbiota and promote the growth of probiotics (56, 57). Numerous studies have shown that most EMPs are fermented by the intestinal microbiota, resulting in the generation of short-chain fatty acids (SCFAs) and alteration of the microbiota composition (Table 3). For instance, after simulated digestion, the *Pleurotus eryngii polysaccharide* (PEP) remained structurally intact with maintained molecular weight and was effectively utilized and broken down by the intestinal microbiota through fermentation, leading to the production of various SCFAs (62). Conversely, the molecular weight of *Oudemansiella radicata* polysaccharides (ORP) decreased during simulated digestion, but its overall structure remained intact. Additionally, no free monosaccharides were detected, implying that ORP is indigestible (64).

**Table 3:** Digestive characteristics and fermentation of EMPs *in vivo*.

**Source of EMPs**	***In vivo* model**	**Digestive characteristics**	**Gut microbiota regulation**	**SCFAs**	**References**
*Hericium erinaceus*	Saliva-gastrointestinal digestion and prebiotic impact.	The substance remains undigested during simulated exposure to saliva, gastric juice, and small intestinal juice, but ultimately reaches the large intestine where it undergoes degradation by intestinal flora.	*Bifidobacterium, Faecalibacterium, Blautia, Butyricicoccus, Lactobacillus* ↑; *Escherichia-Shigella, Klebsiella, Enterobacter* ↓	Acetic acid, propionic acid ↑	(58)
*Clitocybe squamulose*	Saliva-gastrointestinal digestion and fermentation.	The substance is resistant to hydrolysis by saliva, undergoes a reduction in molecular weight during gastric digestion, and does not affect the structural characteristics of the functional groups of the polysaccharide.	*Bacteroides, Parabacteroides* ↑; *Escherichia-shigella* ↓; the ratio of *Firmicutes* to *Bacteroidetes* (F/B) ↓	Acetic acid, propionic acid, isobutyric, hexanoic acid ↑	(59)
*Ramaria flava*	Saliva-gastrointestinal digestion and prebiotic effects observed.	After 4 h of exposure to gastric juice, the substance underwent a 0.7% degradation, and a 0.90% degradation in intestinal juice.	Stimulates the growth of *Lactobacillus rhamnosus* and regulates pH.	Acetic acid, propionic acid, isobutyric, hexanoic acid ↑	(60)
*Tremella fuciformis*	Saliva-gastrointestinal digestion and fermentation.	No changes observed in the reducing sugar content, chemical composition, rheological properties, molecular weight, or constituent monosaccharides.	*Phascolarctobacterium, Bacteroides, Lachnoclostridium* ↑	acetic, propionic, n-butyric, n-valeric acids ↑	(61)
*Pleurotus eryngii*	Saliva-gastrointestinal digestion and fermentation.	The molecular weight remained unchanged, and the overall structure was not destroyed.	*Enterococcus, Streptococcus, Clostridium* ↑; *Escherichia Shigella, Desulfovibrio, Desulfovibrio* ↓	acetic acid, propionic acid ↑	(62)
*Sparassis crispa*	Saliva-gastrointestinal digestion and fermentation.	No changes observed in the reducing sugar content, no free monosaccharides, and no significantly damaged structure.	*Prevotella 9, Dialister, Megamonas, Megasphaera* ↑; *Escherichia Shigella* ↓	acetate, propionate, butyrate ↑	(63)
*Oudemansiella radicata*	Saliva-gastrointestinal digestion and fermentation.	No free monosaccharides observed, and the overall structure was not destroyed.	*Bacteroides, Parabacteroides* ↑	acetic acid, propionic acid, n-butyric acid ↑	(64)
*Ganoderma lucidum* spore	*in vitro* hydrolysis using artificial human gastric juice and fermentation.	Resistance to hydrolysis by artificial human gastric juice.	*Ruminococcaceae, Bifidobacteriaceae, Lactobacillaceae* ↑; *Enterobacteriaceae, Lachnospiraceae* ↓	Nm	(65)
*Lentinula edodes*	Saliva-gastrointestinal digestion and fermentation.	Reducing sugar content increased, while the molecular weight remained relatively unchanged.	*Bacteroides* ↑	propionic acid, butyric acid ↑	(66)
*Ganoderma lucidum*	*In vitro* fermentation.	Nm	*Bacteroides* , *Shigella* ↑; *Peptostreptococcus, Phascolarctobacterium, Fusobacterium, Lachnospiraceae Clostridium* ↓; The ratio of *Firmicutes* to *Bacteroidetes* (F/B) ↓	acetic, propionic, butyric acids ↑	(67)

Nm, not mentioned; ↑, increase; ↓, reduction.

### 3.2. EMPs impact the composition of the gut microbiota community

EMPs, which are difficult to digest and absorb, can function as a carbon source for the intestinal microbiota. This, in turn, can enhance human intestinal health and preserve physiological activity by promoting diversity and regulating the composition of gut microbiota. Research has demonstrated that the protective effects of EMPs on intestinal microbiota regulation are diverse. In terms of gut microbiota composition, The intake of EMPs can stimulate the growth of beneficial bacteria while suppressing the harmful ones, which contributes to a more balanced microbiota composition (68). In terms of gut microbiota function, EMPs can enhance CAZymes activity, increase SCFAs production, reduce proinflammatory factor expression, and boost tight junction protein expression, thereby promoting the overall intestinal health of the organism (69). *In vitro* fermentation studies have shown that *Pleurotus eryngii* polysaccharide (PEP) altered gut microbiota composition by boosting *Firmicutes* and reducing *Proteobacteria* and *Bacteroidetes* (62). *Tremella fuciformi* polysaccharide was found to be significantly consumed by the colonic microbiota in human feces, stimulating the growth of *Phascolarctobacterium, Bacteroides*, and *Lachnoclostridium* (61). Meanwhile, *Cyclocybe cylindracea, Pleurotus eryngii*, and *Pleurotus ostreatus* polysaccharides positively impacted gut microbiota composition, increasing populations of *F. prausnitzii* and *Bifidobacterium*spp (70). *Flammulina velutipes* polysaccharides elevated the *Bacteroidetes* to *Firmicutes* ratio and fostered the growth of beneficial gut microbiota, particularly *Bifidobacterium* and *Bacteroides* (71). Although *in vitro* studies are confined to specific types of gut microbiota bacteria, it is noteworthy that EMPs have a considerable influence on the diversity, richness, and overall composition of the gut microbiota. Additionally, EMPs from various sources have different impacts on gut microbiota modulation, which could be due to structural variations in the EMPs (72, 73).

Besides, research from both animal models and clinical trials has shown that the consumption of EMPs can modify the composition of the gut microbiota. Animal studies have demonstrated that *Agrocybe cylindracea* Polysaccharides can favorably modify the gut microbiota and related metabolites, reducing levels of *Desulfovibrio* and increasing abundance of *Parabacteroides* to prevent diet-induced obesity in mice (74). *Tremella fuciformis* polysaccharide (TPs) can substantially increase the diversity of the gut microbiota and restore the relative abundance of certain bacterial species, such as *Odoribacter, Lactobacillus, Marinifilaceae*, and *Ruminococcaceae*. This finding suggests that TPs may exert a protective effect on dextran sulfate sodium (DSS)-induced colitis in mice (75). Furthermore, in a clinical trial, a β-D-glucan-enriched extract derived from *Lentinula edodes* was found to raise the relative abundance of *Ruminococcaceae UCG-014, Erysipelotrichaceae UCG-003, Akkermansia*, and *Subdoligranulum*, thereby influencing lipid metabolism (76).

In summary, the supplementation of EMPs can alter the composition of the gut microbiota, enhance diversity and richness, and raise the abundance of various species, all of which have important health implications. Additionally, EMPs can restrict the growth of harmful microorganisms. There are several reasons behind these effects. Firstly, gut microbiota ferment EMPs into short-chain fatty acids (SCFAs) as end-products, which lower the intestinal pH and provide a more favorable environment for the growth of beneficial bacteria (66, 76). For instance, *Firmicutes* are more tolerant to weakly acidic environments than *Bacteroidetes* (77). Microorganisms specializing in polysaccharide degradation become dominant by breaking down polysaccharides (78). Moreover, some microorganisms can use the metabolites produced during the degradation of EMPs by other microorganisms as a carbon and energy source to promote their growth (79). For example, multi-species symbiotic cross-feeding can occur between *Enterobacteriaceae* and *Bacteroidales* spp., leading to synergistic development of mixed communities *in vitro* (80). *Ruminococcus bromii* can produce fermentation products by fermenting resistant starch to support the growth of other symbiotic bacteria (81). Overall, while it is evident that EMPs play a role in regulating gut microecology, the precise mechanisms by which they affect microbial growth and the pathways involved are still unclear and require further investigation. In addition, mushrooms may be a source of toxic heavy metals that may have adverse health effects (82). For example, the report on heavy metal concentrations in wild edible mushrooms in Yunnan Province (China) showed that heavy metal contamination in wild edible mushrooms is a serious problem (83). More studies are recommended to elucidate possible procedures for the accumulation of toxic heavy metals in edible mushrooms.

### 3.3. EMPs influence SCFAs production

The gut microbiota can produce energy for its growth through the fermentation of EMPs. This process generates a mixture of gases, including CO_2_, methane, and hydrogen, as well as short-chain fatty acids (SCFAs) such as acetic, propionate, and butyrate. Studies have demonstrated that SCFAs can reach other tissues and organs through the circulatory system, contributing to various health-promoting effects. These molecules modulate the intestinal immune system to maintain homeostasis and regulate the production of hormones such as Glucagon Peptide 1 (GLP-1) and insulin, through their interactions with L-endocrine cells and small intestine endothelial cells (84). SCFAs also activate G-protein-coupled receptor (GPCR) signaling pathways, inducing the transcription of factors such as hypoxia-inducible factor 1 (HIF-1), signal transducer and activator of transcription 3 (STAT3), and specificity protein 1 (SP1), thereby enhancing epithelial barrier function, boosting the production of antimicrobial peptides, and reducing inflammation (85). Furthermore, they reduce the activity of histone acetyltransferases and histone deacetylases, which control gene expression, and prevent the maturation of monocytes into dendritic cells and macrophages, along with their production of inflammatory cytokines (86). SCFAs have also been shown to act as ligands for the free fatty acid receptor (FFAR), influencing the host's immunological activity (87). Therefore, SCFAs have a broad range of effects on host metabolism, proliferation, and differentiation, serving as signaling molecules.

Studies have shown that SCFAs are the primary byproducts of gut microbiota fermentation of EMPs and play a critical role in maintaining overall health. For instance, polysaccharides derived from *Flammulina velutipes* have been shown to increase the levels of acetic acid, propionic acid, and butyric acid, which can enhance the immunity of immunocompromised mice (88). The polysaccharides from *Ganoderma lucidum* have also been observed to increase the concentration of SCFAs in human gastrointestinal simulations, specifically acetic acid, propionic acid, and n-butyrate (89). Furthermore, polysaccharides from *Sparassis crispa* have been found to regulate blood sugar levels through modulation of SCFAs concentration (90). Therefore, the health benefits of EMPs can be attributed to the type and amount of SCFAs produced in the gut.

### 3.4. Effect of molecular weight, polysaccharide composition, and structure of EMPs on intestinal microbiota

The structural features of EMPs have significant impacts on their biological activities and their regulatory effects on the gut microbiota (Table 4). Specifically, the molecular weight of EMPs can affect their absorption and metabolism in the gut. Research has shown that EMPs with lower molecular weights are more readily metabolized by gut microbiota, leading to higher production of short-chain fatty acids (SCFAs), such as propionate, butyrate, and acetate, while EMPs with higher molecular weights are less readily metabolized, resulting in lower SCFA production (108–110). For example, low molecular weight polysaccharides (<10 kDa) from *Ganoderma lucidum* have better fermentation and higher gas production ability, stimulating the growth of intestinal bacteria quickly. On the other hand, high molecular weight polysaccharides (>100 kDa) are more difficult to be fermented by intestinal bacteria and have a longer residence time in the intestines, resulting in a more prolonged effect on the intestinal microbiota (110). Furthermore, the monosaccharide composition of EMPs also plays a crucial role in regulating the gut microbiota. For instance, glucose, xylose, and galactose are among the most common monosaccharides found in EMPs, with glucose being one of the most prevalent. Glucose has been found to promote the growth and metabolism of gut microbiota, thereby maintaining gut health (79). Research has indicated that glucose can stimulate the metabolic activity of gut microbiota, increase the growth of beneficial bacteria, and promote the production of metabolites, thereby enhancing the immune function of the gut and protecting the gut mucosa (111). Although xylose and galactose are present in relatively low amounts in EMPs, their effects on the gut microbiota cannot be ignored. Xylose can promote the growth of probiotics and protect the gut mucosa by increasing the production of beneficial metabolites (112). Galactose, on the other hand, can inhibit the growth of harmful bacteria, reducing the production of harmful metabolites and benefiting gut health (113). For example, A polysaccharide from *Pleurotus eryngii*, rich in glucose (78.32%), galactose (8.47%), and mannose (9.43%), promoted the relative abundance of *Firmicutes* and reduced that of *Bacteroidetes* and *Proteobacteria*, while increasing the production of acetic and propionic acids (62). A polysaccharide from *Hericium erinaceus*, composed of fructose, mannose, glucose and galactose, enhanced the abundance of SCFA-producing bacteria (58). In addition, the length and branching patterns of EMPs can also affect their degradation and absorption in the gut (2). Studies have indicated that shorter chain lengths and fewer branching patterns can enhance the absorption and utilization of EMPs in the gut, resulting in better regulatory effects (18). Additionally, the length and branching patterns of EMPs can also influence their metabolites in the gut, which in turn affect the growth and metabolism of gut microbiota (114). Zhang et al. obtained *Sparassis crispa* polysaccharides with main chain structures of (1 → 6)-α-D-Galp, (1 → 6)-β-D-Glcp and (1 → 3)-β-D-Glcp and side chain structures of (1 → 4)-β-D-Glcp, (1 → 3)-β-D-Glcp, T-α-L-Fucp and T-β-D-Glcp can promote the production of short-chain fatty acids and the abundance of probiotics, such as *Dialister* and *Megasphaera* (63, 98). Therefore, in order to fully realize the regulatory effects of EMPs, further research is needed to explore the relationships between their structural features and gut microbiota metabolism, as well as the impacts of different mushroom species and polysaccharide sources on their regulatory effects. This will provide a more robust scientific basis for the application of EMPs.

**Table 4:** Effect of the structure of EMPs on gut microbiota.

**Source of EMPs**	**Monosaccharide composition**	**Molecular weight (Da)**	**Structure characteristics**	**Gut microbiota regulation**	**References**
*Agaricus bisporus*	Ribose, rhamnose, arabinose, xylose, mannose, glucose, and galactose at a molar ratio of 2.08: 4.61: 2.45: 22.25: 36.45: 89.22: 1.55.	7.84 × 10^5^	α-pyran polysaccharide composed of 1 → 2 and 1 → 4 glycosidic bonds.	Promoted the growth of beneficial bacteria, including *Prevotella, Megamonas*, and *Bacteroides*.	(72)
*Oudemansiella radicata*	Glucose (59.19%), galactose (22.63%) mannose (7.76%), fucose (6.46%), xylose (3.97%).	5.2 × 10^3^	α-pyran polysaccharide composed of 1 → 2 and 1 → 4 glycosidic bonds.	Promote the growth of *Bifidobacterium* and *Lactobacillus*, increase the production of SCFAs.	(64)
*Agaricus bisporus*	D-glucose, D-mannose, D-galactose and D-xylose in the molar ratio of 2.25:2.00: 0.35:0.20.	5.17 × 10^4^	→ 6)-β-D- Gluc- (1 → 4)-α-D- Manp(1 → 6)-β-D-Glcp- (1 → 6)-β-D-Glucp-(1 → .	Increase α-diversity, the short-chain fatty acid (SCFA) level and the abundance of beneficial genera, such as *Bacteroides* and *Parabacteroides*.	(91, 92)
*Flammulina velutiper*	D-galactose, D-mannose, L-fucose, and D-glucose at molar ratio of 1.9:1.2:1:2.5.	1.50 × 10^4^	→ 2)-α-D-Galp-(1 → 4)-α-D-Galp-(1 → 6)-α-D-G1cp-(1 → 3)-β-D-Glcp-(1 → .	Increase the abundance of *Bacteroides*, decrease the abundance of *Desulfovibrionales* and *Clostridium*.	(93, 94)
*Grifola frondosa*	Mannose, rhamnose, glucose, galactose, fucose at molar ratio of 25.49: 5.18: 27.59: 15.02: 9.92.	1.58 × 10^7^	(1 → 4)-linked and (1 → 6)-linked α-d-glucopyranosyl, and (1 → 3,6)-linked α-d-mannopyranosyl residues and the branches consisted of (1 → 6)-linked α-d-galactopyranosyl and t-l-rhamnopyranosyl residues.	Increase the relative abundances of *Alistipes* and reduce *Streptococcus, Enterococcus, Staphylococcus* and *Aerococcus*.	(95, 96)
*Inonotus obliquus*	Mannose (14.19%), rhamnose (6.46%), glucuronic acid (3.76%), xylose (9.07%), arabinose (3.84%), fucose (2.98%), galacturonic acid (9.15%), glucose (29.85%), and galactose (20.70%).	3.73 × 10^5^	α-pyranose polysaccharide.	Increased the abundance of *Akkermansia* and *Lactobacillus*.	(95)
*Sparassis crispa*	Glucose, galactose, fucose, and mannose in a molar ratio of 52.10: 31.10: 15.04: 1.76.	1.36 × 10^4^	(1 → 6)-α-D-Galp, (1 → 6)-β-D-Glcp, (1 → 3)-β-D-Glcp, (1 → 2,6)-α-D-Galp and (1 → 3,6)-β-D-Glcp.	Increase the abundance of *Prevotella 9, Dialister, Megamonas* and *Megasphaera*.	(63, 97, 98)
*L. edodes*	Glucose (64.7), mannose (16.9), galactose (11.4), Xylose (5.0), arabinose (2.0).	2.3 × 10^4^	β-1,3-linked glucan with β-1,6 branches.	Increases the abundance of *Proteobacteria, Alistipes* and *Bacteroides acidifaciens*.	(99, 100)
*H. erinaceus*	Xylose (7.8%), ribose (2.7 %), glucose (68.4%), arabinose (11.3%), galactose (2.5%), mannose (5.2%).	1.8 × 10^4^ - 1.2 × 10^6^	β-(1 → 3)-glucan with β-(1 → 6)-branches; some contain α-(1 → 6)-linked galactose residues.	Increase the diversity and abundance of beneficial bacteria, such as *Bifidobacterium* and *Lactobacillus*.	(101–103)
*Pleurotus abieticola*	Fucose (1.73%), galactose (49.66%), glucose (12.00%), mannose (36.60%).	1.72 × 10^4^	→ 2,6)-α-D-Galp-(1 → , → 6)-α-D-Galp-(1 → and → 3)-β-D-Glcp-(1 → residues, and branches mainly with β-D-Manp-(1 → and β-D-Manp-(1 → 6)-α-D-Galp-(1 → and linkages were attached at the C-2 of the → 2,6)-α-D-Galp-(1 → residue.	Increase the abundance of *Prevotella, Alistipes, Coprococcus* and *Oscillospira*.	(104)
*Lyophyllum decastes*	Mannose, glucose, galactose, and fucose at a similar ratio (1:2.38:2.58:0.73 for LDP1-1 and 1:2.33:2.51:0.78 for LDP1-2).	LDP1-1: 5.02 × 10^5^, LDP1-2: 1.13 × 10^6^	3-Fucp, T-Galp, 1,4-Glup, 1,6-Glup, 1,6-Galp, 1,2,6-Manp.	Increase the abundance of *Bacteroides intestinalis* and *Lactobacillus johnsonii*.	(105)
*Dictyophora indusiata*	Glucose (56.2%), mannose (29.7%), galactose (14.1%).	1.81 × 10^4^	→ 1)-Glc-(6 → 1)-Man-(3,6 → 1)-Xyl-(5 → 1)-Gal-(3 → 1)-Gal-(6 → .	Increased the diversity of intestinal microorganisms and the relative abundance of *Lactobacillus*.	(106, 107)

## 4. EMPs regulate intestinal ecological disorders and improve host health

**Figure 3:**
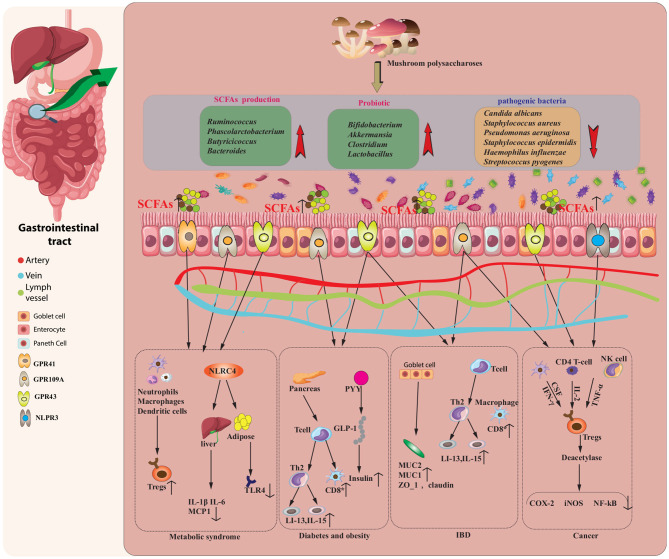
EMPs modulate the composition and function of the intestinal microbiota, as well as its derived short-chain fatty acids (SCFAs), to potentially treat various diseases, including inflammatory metabolic syndrome, diabetes, obesity, bowel disease (IBD), and cancer. EMPs enhance the levels of SCFAs and stimulate the proliferation of probiotics in the gut. These SCFAs modulate immune cells, inhibit pro-inflammatory mediators, and induce anti-inflammatory molecules, ultimately ameliorating metabolic syndrome. The secretion of PYY and GLP-1 and the expansion of regulatory T cells (Tregs) mediated by probiotics contribute to the attenuation of diabetes and obesity, while the adherence of probiotics to the intestinal mucus layer and the induction of anti-inflammatory factors help relieve the symptoms of IBD. Moreover, SCFAs interact with specific receptors to downregulate the expression of pro-inflammatory signaling molecules, resulting in anticancer effects.

Research has demonstrated that EMPs can have substantial positive effects on blood glucose, body weight, insulin resistance, inflammation, endotoxemia, and gut ecological dysregulation, while preserving the integrity of the gut barrier. For intestinal dysbiosis, EMPs promote the proliferation of beneficial bacteria, inhibit the proliferation of pathogenic bacteria, and increase the concentration of SCFAs in the intestine (2). Beneficial bacteria, such as *Lactobacillus, Bifidobacteria, Streptococcus*, and *Bacillus coagulans*, establish a symbiotic relationship with the host and constitute the primary bio-antagonistic and bio-barrier microbiota. They perform essential immune, metabolic, and nutritional functions within the host (115). On the other hand, harmful bacteria, including *Shigella flexneri, Citrobacter rodentium, Listeria monocytogenes*, and *Salmonella enterica*, are typically non-pathogenic to the host under normal circumstances. However, if their populations exceed a certain threshold, they can instigate infections and result in disease (116). As a result, EMPs can be considered a nutritious and potentially beneficial bioactive food source for promoting overall health, as summarized in Figure 3 and Tables 5, 6.

**Table 5:** Health improvement of EMPs through gut microbiota regulation in different disease models.

**Source of EMPs**	**Models**	**Gut microbiota modulation**	**Health improvements**	**References**
*Polyporus umbellatus*	DSS-induced colitis ICR mice.	*Lactobacillus, Clostridium, Lachnospiraceae* ↑; *Alistipes_finegoldii* ↓	Increased diversity of gut microbiota mitigated colonic injury, while also promoting splenic lymphocyte proliferation, enhancing serum hemolysin synthesis, and increasing peripheral phagocytosis and NK-cell activity.	(117)
*Helvella leucopus*	DSS-induced colitis C57BL/6 male mice.	*Lactobacillus, Akkermansia* ↑; *Lachnospiraceae genera, Lachonospiraceae_NK4A136, Lachnospiraceae_unclassified* ↓	A dose-dependent downregulation for pro-inflammatory cytokines (IL-6, IL-1β, and TNF-α), as well as for pro-inflammatory mediators (COX-2 and iNOS), while the expression of anti-inflammatory cytokine IL-10 was upregulated.	(118)
*Lyophyllum decastes*	High-fat diet (HFD)-induced obese C57BL/6J male mice.	*L. johnsonii, B. sartorii, B. intestinalis* ↑; *Firmicutes/Bacteroidetes* (F/B) ↓	Remodeling of the gut microbiota and activation of the TGR5 signaling pathway can improve HFD-induced obesity, hyperlipidemia, and inflammation.	(105)
*Pleurotus ostreatus*	High-fat diet (HFD)-induced obese C57BL/6J male mice.	*Oscillospira, Lactobacillus, Bifidobacterium* ↑; *Bacteroides, Roseburia* ↓	Prevented obesity, maintained glucose homeostasis, and had a beneficial impact on the gut microbiota.	(119)
*Cordyceps militaris*	High-fat diet (HFD)-induced obese C57BL/6J male mice.	*Akkermansia* ↑; *Dorea, Lactobacillus, Clostridium, Ruminococcus* ↓	Ameliorated fat accumulation, dyslipidemia, inflammation, and dysbiosis of the gut microbiota.	(120)
*Cordyceps militaris*	High-fat/high-sucrose diet (HFSD) induced obese C57BL/6J male mice.	*Akkermansia, Lachnospiraceae_Eubacterium* ↑; *Bacteroides, Parabacteroides, Blautia* ↓	Decreased blood sugar and serum lipid levels and improved intestinal dysbiosis.	(121)
*Cordyceps militaris*	High-fat diet (HFD)-induced obese C57BL/6J male mice.	*Alloprevotella, Parabacteroides, Butyricimonas, Alistipes* ↑; *Negativebacillus* ↓	Led to a reduction in body weight and fat accumulation, as well as a decrease in pro-inflammatory cytokine levels, an improvement in glucose tolerance, and a restoration of gut barrier function.	(122)
*Pleurotus eryngii*	High-fat diet (HFD)-induced obese C57BL/6J male mice.	*Anaerostipes, Clostridium, Lactococcus* ↑; *Roseburia* ↓	Suppressed weight gain and fat accumulation, improved glucose tolerance, and decreased LDL cholesterol levels.	(123)
*Ganoderma lucidum*	High-fat diet (HFD)-induced obese Wistar rats.	*Alloprevotella, Barnesiella, Parabacteroides, Bacteroides Bacteroidales S24-7, Alistipe* ↑; *Blautia, Roseburia, Enterorhabdus* ↓; butyric acid ↑	Suppressed hepatic lipid accumulation and steatosis, while promoting fecal excretion of total bile acids (BAs).	(124)
*Grifola frondosa*	High-fat diet (HFD)-exacerbated hyperlipidemic and hypercholesterolemic Wistar rats.	*Helicobater, Intestinimonas, Barnesiella, Parasutterella, Ruminococcus, Flavonifracter* ↑; *Clostridium-XVIII, Butyricicoccus, Turicibacter* ↓	Gut microbial phylotypes can be modulated to improve lipid metabolic disorders, while also regulating the expression of genes involved in hepatic lipid and cholesterol metabolism.	(125)
*Ganoderma lucidum*	High-fat diet (HFD) induced hyperlipidemic and hypercholesterolemic Wistar rats.	*Alistipes, Prevotella, Alloprevotella, Defluviitalea* ↑; *Turicibacter, Phascolarctobacterium* ↓	Resulted in a decrease in serum levels of total triglyceride (TG), total cholesterol (TC), low-density lipoprotein cholesterol (LDL-C), alanine transaminase (ALT), and free fatty acids (FFA), as well as a reduction in fasting blood glucose (FBG) and inhibition of hepatic steatosis.	(126)
*Agrocybe cylindracea*	High-fat diet (HFD)-induced obese C57BL/6J male mice.	*Bacteroides, Parabacteroides, Butyricimonas, Dubosiella* ↑; *Desulfovibrio, Oscillibacter* ↓; The ratio of *Firmicutes/Bacteroidetes* (F/B) ↓	Reduced body weight, adipose tissue accumulation, improved insulin resistance, lowered lipid levels, improved liver injuries, and restored gut dysbiosis.	(74)
*Ganoderma lucidum*	High-fat diet (HFD)-induced obese C57BL/6J male mice.	*Bifidobacterium choerinum, Bacteroides chinchillae* ↑	Led to modulation of the gut microbiota, improved gut barrier function, increased production of SCFAs, activation of GPR43, and inhibition of the TLR4/Myd88/NF-κB signaling pathway.	(69)

↑, increase; ↓, reduction.

**Table 6:** Health improvement of EMPs through gut microbiota regulation in different disease models.

**Source of EMPs**	**Models**	**Gut microbiota modulation**	**Health improvements**	**References**
*Poria cocos*	High-fat diet (HFD)-induced obese C57BL/6J male mice.	*Lactobacillus, Clostridium*; *Lachnospiraceae NK4A136* group, *Alistipes, Ruminococcus1, Faecalibacterium, Desulfovibrio, Mucispirillum* ↓	Ameliorated glucose intolerance and insulin resistance, resulting in decreased levels of blood glucose and insulin. The study also observed suppression of the mRNA expressions of regulators of fatty acid synthesis and pro-inflammatory cytokines in epididymal fat.	(127)
*Auricularia auricula*	High-fat diet-induced hyperlipidemia male Sprague-Dawley rats.	*Roseburia, Flavonifractor, Clostridium IV* ↑; acetate, propionate, butyrate ↑	Ameliorated lipid metabolism, resulting in a reduction in the levels of total cholesterol and low-density lipoprotein cholesterol.	(128)
*Ganoderma frondosa*	Streptozotocin (SZT)-induced type 2 diabetic Male ICR mice fed with a high-sucrose/high-fat diet.	*Blautia, Bacteroides Dehalobacterium, Parabacteroides* ↑; *Proteus, Aerococcus, Ruminococcus, Corynebactrium* ↓	Resulted in a decrease in fasting blood glucose levels, improved oral glucose tolerance, and alleviated insulin resistance, thereby protecting against liver and kidney injury. These effects were achieved by regulating the IRS1/PI3K and JNK signaling pathways.	(129)
*Grifola frondosa*	High-fat diet (HFD) and streptozotocin (STZ)-induced diabetic male Kunming mice.	*Alistipes* ↑; *Streptococcus, Enterococcus, Staphylococcus, Aerococcus* ↓	Regulated the mRNA expression levels of genes responsible for hepatic glucose and lipid metabolism, including cholesterol 7α-hydroxylase and bile salt export pump, to prevent hyperglycemia and hyperlipidemia.	(95)
*Ganoderma lucidum*	High-fat diet (HFD) and streptozotocin (STZ) induced diabetic male Kunming mice.	*Lactobacillus, Bacteroides, Ruminococcaceae* ↑; The ratio of *Firmicutes/Bacteroidetes* (F/B) ↓	Repaired islet cells and increased insulin secretion, promoted the synthesis and storage of glycogen in the liver, and improved the activities of antioxidant enzymes and insulin resistance. As a result, the homeostasis model assessment for insulin resistance (HOMA-IR) declined.	(130)
*Armillariella tabescens*	High-fat diet (HFD) and streptozotocin (STZ) induced diabetic male C57BL/6J mice.	*Lactobacillus, Akkermansia* ↑; *Proteobacteria* ↓; the ratio of *Firmicutes/Bacteroidetes* ↓	Modulated the composition of the intestinal microbiota, improved the intestinal barrier function, reduced the lipopolysaccharide (LPS) content and systemic inflammation, and ultimately alleviated renal damage.	(131)
*Auricularia auricular-judae*	Dextran sulfate sodium (DSS)-induced colitis half male and half female BALB/C mice.	*Bacteroidetes* ↑; *Ruminococcus, Deferribacteres, Actinobacteria* ↓	Ameliorated weight loss, colon shortening, mucosal inflammation, damage to the intestinal barrier, and dysbiosis of gut microbiota.	(132)
*Flammuliana velutipes*	DSS-induced colitis SD male rats.	the ratio of *Firmicutes/Bacteroidetes* ↑; *Lachnospiraceae, Bacteroidales S24-7* ↑; Butyric, isovaleric, valeric acid ↑	Regulated colonic microbial dysbiosis and promoted the levels of caecal SCFAs, leading to the down-regulation of the TLR4/NF-κB signaling pathway.	(133)
*Ganoderma lucidum*	AOM/DSS-induced colitis in C57BL/6 mice.	*Bifidobacterium, Lactobacillus* ↑; *Lachnoclostridium, Oscillibacter, Desulfovibrio, Alistipes, Parasutterella* ↓; acetate, propionate, butyrate ↑	Ameliorated microbiota dysbiosis, increased the production of short-chain fatty acids, and alleviated endotoxemia by inhibiting the TLR4/MyD88/NF-κB signaling pathway.	(134)
*Tremella fuciformis*	DSS-induced colitis in C57BL/6 mice.	*Lactobacillus, Ruminococcaceae, Odoribacter, Helicobacter, Marinifilaceae* ↑	Reducing the activity of colonic myeloperoxidase and serum diamine oxidase (DAO), lowering the concentration of D-lactate, and alleviating colonic tissue damage can be achieved through the stimulation of Foxp3+ T cells, which promote the production of anti-inflammatory cytokines.	(75)

↑, increase; ↓, reduction.

### 4.1. Metabolic disorders

Metabolic diseases encompass a range of conditions affecting the metabolism of the human body. Research on metabolic syndrome populations and animal models has revealed that gut ecological dysbiosis often results in a decrease in the abundance of *probiotics*, an increase in opportunistic and pathogenic bacteria, and a reduction in microbial diversity (135). Imbalances in the intestinal ecosystem can cause a reduction in the expression of tight junction proteins in the intestine, resulting in an increase of intestinal permeability, impaired intestinal integrity, and the release of bacterial debris and endotoxins from the gut into the bloodstream. This triggers the activation of the NF-κB and NLRP3 signaling pathways in inflammatory vesicles, resulting in elevated levels of pro-inflammatory factors such as IL-1, IL-6, and TNF-α, ultimately leading to chronic low-grade inflammation throughout the body (136). Additionally, activation of the NF-κB signaling pathway increases the levels of protein phosphatase 1B (PTP1B), which dephosphorylates the insulin receptor or its substrate, hindering the insulin receptor's ability to bind with insulin, and causing insulin resistance (137). Dysbiosis in the intestinal tract also impairs the synthesis of vitamins K and B, folic acid, neurotransmitters, SCFAs, and other bioactive components by gut microbes (138). Therefore, imbalances in the gut microbiota are a key contributor to the onset and expression of metabolic disorders.

Studies have indicated that the consumption of EMPs has a positive impact on metabolic disorders. In animal models, such as dietary-induced obese mice, a polysaccharide from *Ganoderma lucidum* (BSGLP) containing (1 → 3)-β-D-Glcp, (1 → 3,6)-β-D-Glcp, (1 → 6)-β-D-Glcp, and terminal-β-D-Glcp moieties has been demonstrated to restore balance to the gut microbiota and mitigate dysbiosis, leading to a reduction in fat accumulation and a decrease in inflammation (69). Similar results have been observed with *Dictyophora indusiata* polysaccharides consisting of glucose 59.84%, mannose 23.55% and galactose 12.95% which mitigated hepatic steatosis, hyperlipidemia, hyperglycemia, and LPS-induced inflammation in mice with high-fat diet-induced obesity (DIO) by reducing the abundance of *Bacilli, Gammaproteobacteria*, and increasing abundance of *Bacteroidiaas* (139). Supplementation with *Cordyceps militaris* Polysaccharides containing 1, 3-branched-galactomannoglucan with a linear backbone of (1 → 4)-linked α-D-Glcp in mice on a high-fat diet led to reduced body weight, pro-inflammatory cytokine levels, and fat accumulation, as well as improved glucose tolerance and intestinal barrier function. This was shown to be a result of reversing intestinal dysbiosis, as evidenced by an increased population of *Alloprevotella, Butyricimonas, Parabacteroides*, and *Alistipes* (122). Supplementation with *Ganoderma lucidum* polysaccharides consisting of arabinose, galactose, glucose, xylose, mannose, ribose, and rhamnose with molar percentages of 5.32%, 5.47%, 57.63%, 0.84%, 25.41%, 1.95%, and 3.38%, respectively, and molecular weight of 15900 Da improved insulin secretion and repaired islet cells, stimulated glycogen synthesis and storage in the liver, and enhanced antioxidant enzyme activities and insulin sensitivity in diabetic mice induced by a high-fat diet (HFD) and streptozotocin (STZ) by elevating the abundance of *Lactobacillus, Bacteroides*, and *Ruminococcaceae* (130). The polysaccharide from *Ganoderma lucidum* with a molecular weight of 13.7 kDa, mannose, glucose, galactose, rhamnose, and arabinose in a molar ratio of 3.16:16.17:3.74:1.65:1 effectively reduced fasting blood glucose and insulin levels and improved gut microbiome imbalances by decreasing the levels of *Aerococcus, Corynebacterium, Proteus*, and *Ruminococcus* (140). Polysaccharide extracted from *Phellinus linteus* with the backbone of → 3)-β-d-Glcp-(1 → and → 6)-β-d-Glcp-(1 → has been shown to effectively reduce fasting blood glucose levels in mice. This is achieved by altering liver phospholipid metabolism and improving insulin signaling, as well as increasing the abundance of *Porphyromonas* (141). In general, EMPs have the potential to regulate gut microbiota by promoting the growth of beneficial bacteria and inhibiting that of harmful bacteria, which could improve gut health and prevent or treat metabolic disorders.

### 4.2. Inflammatory bowel disease

Inflammatory bowel disease (IBD), encompassing ulcerative colitis (UC) and Crohn's disease (CD), is marked by persistent inflammation in the gastrointestinal tract (142). Symptoms of IBD include diarrhea, bloating, abdominal pain, blood in stools, weight loss, and discomfort. Research suggests that IBD arises from a genetically susceptible individual's misguided inflammatory response to the gut microbiome (143). The most prevalent imbalance in the intestinal flora among IBD patients is a decrease in *Bifidobacterium adolescentis, prausnitzii, Dialister invisus, Faecalibacterium* and an unnamed member of *Clostridium cluster XIVa*, and an increase in *Ruminococcus gnavus* (144, 145). In addition to intestinal ecological dysbiosis, metabolites derived from gut bacteria, such as short-chain fatty acids (SCFAs), bile acids (BAs) and tryptophan metabolites, also play a crucial role in the development and progression of IBD. The latest extensive and comprehensive study found that gut microbiome metabolites impact host immunity, mucosal health, homeostasis, and energy metabolism (146). As such, the composition and function of the intestinal microbiota are crucial factors that impact the progression of IBD.

Medical treatments for IBD mainly aim to control inflammation and prevent the progression of the disease. There are three main types of conventional drugs that are commonly used in IBD treatment, including aminosalicylic acid derivatives, glucocorticoids, and immunosuppressive drugs (147). However, prolonged use of these drugs can lead to reduced immunity, and increase the risk of electrolyte imbalances, peptic ulcers, cataracts, and osteoporosis (148). Recent studies have shown that EMPs can improve intestinal health with minimal adverse effects. EMPs, as a natural immunomodulator, are effective in treating and preventing IBD by regulating gut bacteria and upregulating the expression of tight junction proteins (TJs), such as junctional adhesion molecules, cytosolic scaffold proteins, occludin, and intracellular zonulae occludens like ZO-1. For instance, high doses of polysaccharides from *Tremella fuciformis* have the potential to improve gut health by increasing the diversity of the gut microbiome, restoring the relative abundance of specific bacteria such as *Helicobacter, Odoribacter, Lactobacillus, Marinifilaceae*, and *Ruminococcaceae*, and activating Foxp3+ T cells to produce anti-inflammatory cytokines. This was observed in mice with colitis induced by dextran sulfate sodium, and the treatment showed minimal side effects (75). A polysaccharide from *Flammulina velutipes* has been shown to alleviate colitis by controlling colonic microbial dysbiosis, increasing short-chain fatty acids, and inhibiting the TLR4-NF-κB signaling pathway (133). The polysaccharide from *Dictyophora indusiata* consisting of 59.84% glucose, 23.55% mannose, and 12.95% galactose has been shown to improve gut microbiota composition and intestinal barrier function by increasing the expression of mucins and tight junction proteins, suppressing harmful bacteria such as *Gammaproteobacteria, Proteobacteria, Bacteroidaceae, Bacteroides*, and *Enterobacteriaceae*, and enhancing beneficial bacteria such as *Lactobacillus acidophilus* (149). Polysaccharides isolated from sporoderm-removed fragments of *Ganoderma lucidum* consisting of arabinose (4.19%), mannose (15.69%), glucose (78.15%), and galactose (1.97%) have been shown to mitigate colitis induced by Azoxymethane/Dextran Sodium Sulfate by improving gut dysbiosis, increasing short-chain fatty acid production, and suppressing TLR4/MyD88/NF-κB signaling to alleviate endotoxemia (134). Yu et al. (150) demonstrated that polysaccharides from *Porphyra haitanensis* improved the integrity of the colonic mucosal barrier by upregulating tight junction proteins, augmenting the mucus layer and its secretion, and regulating the gut microbial community, enriching beneficial bacteria such as *Bacteroides, Muribaculum*, and *Lactobacillus* species, thus mitigating DSS-induced colonic injury. In general, EMPs may alleviate IBD by regulating gut microbiota and repairing the damaged intestinal barrier, creating an environment conducive to the growth of probiotics and suppressing potentially pathogenic bacteria, and modulating TLR4 and NF-κB signaling pathways to reduce intestinal inflammation.

### 4.3. Tumor/Cancer

Microbial commensal dysbiosis, responsible for 20% of tumorigenesis and a greater number of cancers, has been linked to microbial pathogens (151). In a healthy gut microbiome, many probiotics and other resident bacteria secrete molecules to fight tumor growth and prevent tumorigenesis. However, when the intestinal flora is dysbiotic, certain bacterial pathogens can grow and proliferate, secreting toxins such as CagA protein from *Helicobacter pylori*, adhesin A (FadA) from *Fusobacterium nucleatum*, and *metalloproteinase toxin* (MP toxin) from *Bacteroides fragilis*, which interfere with the development of the host cell and eventually lead to the onset of cancer (152). A recent study has shown that lung cancer patients experience a significant shift in gut microbiota composition compared to healthy individuals, with pathogens increasing and some probiotic bacteria decreasing (153). Additionally, certain bacteria can also interfere with the host's hormone metabolism. Intestinal dysbiosis also increases the relative abundance of *Clostridium leptum* and *Clostridium coccoides*, leading to increased secretion of bacterial β-glucuronidase enzymes, which promotes cell proliferation in organs such as the endometrium and breast and may contribute to the development of breast cancer (152, 154). During dysbiosis, several microbiota subpopulations may proliferate, producing high quantities of toxins that cause inflammation and cancer.

According to research in *vivo* and clinical tests, consuming EMPs may be a viable and efficient method for cancer prevention and treatment (155). EMPs can induce the proliferation of beneficial bacteria in the gut, thus increasing the ability to combat carcinogens and reducing the nourishment of harmful bacteria. This reduces the amount of harmful substances in the gut, reducing cellular damage and preventing intestinal cancer. In addition, EMPs have been shown to enhance the immune system's activity, which can lead to the inhibition of cancer cell growth. For instance, A polysaccharide extracted from the mushroom *Pleurotus ostreatus* has been demonstrated to decrease tumor cell metastasis and increase survival in mice with H22 malignant ascites. This effect was achieved by downregulating the expression of genes such as Stat3 and Foxp3, as well as releasing immunological factors including TNFα, INFγ, and IL-2 (156). Polysaccharides from *Ganoderma lucidum* have been found to be more effective in alleviating colorectal cancer symptoms than guar gum, as they increase the prevalence of *Akkermansia*, colon length, and downregulate rectal cancer-related genes (157). *Ganoderma lucidum* polysaccharides have demonstrated cancer-preventive and therapeutic functions by dynamically modulating the gut microbiota and host immune responses (158). Studies have also shown that sporoderm-broken fragments of (*G. lucidum*) polysaccharides (BSGLP) were more effective in reducing gastric cancer cell survival than removed fragments of *G. lucidum* (RSGLP). RSGLP induced apoptosis in AGS cells by dramatically increasing cleaved-PARP and decreasing Bcl-2 and pro-caspase-3 expression levels, while enhancing the expression of LC3-II and p62, indicating autophagy was induced and the autophagic flow was disrupted in AGS cells (159). A recent study discovered that polysaccharides from the Sporoderm-Breaking Spore of *Ganoderma lucidum* with a molecular weight of 3659 Da can serve as a natural adjuvant in breast cancer treatment, boosting the number of cytotoxic T cells and helper T cells. The gut microbiota is also affected, with increased levels of *Firmicutes* and *Proteobacteria* and reduced levels of *Actinobacteria, Bacteroidetes*, and *Cyanobacteria*, leading to improved symptoms (160). Additionally, a polysaccharide derived from the spore of *Ganoderma lucidum* when combined with Paclitaxel can act as an adjuvant against Pertussis toxin in breast cancer treatment. This restores gut microbiota dysbiosis, increasing levels of *Ruminococcus* and *Bacteroides* while reducing the presence of cancer-risk genera such as *Odoribacter* and *Desulfovibrio* (161). These studies suggest that EMPs can inhibit tumor proliferation by remodeling the gut microbiota, but the anti-tumor effect of EMPs in regulating intestinal microbiota has been only demonstrated in some EMPs.

### 4.4. Other beneficial effects

In-depth research has revealed that EMPs offer a range of health benefits, including prevention of cardiovascular disease, liver protection, and improvement of neurological symptoms. For instance, treatment with *Auricularia auricula* polysaccharides with monosaccharides composed of glucose (72%), mannose (8%), xylose (10%), and fucose (10%) and containing a pyranose ring has been found to enhance cardiac function due to its potent antioxidant properties (162). Studies have demonstrated that polysaccharides from *Agrocybe aegerita* composed of arabinose, mannose, Galactose, and glucose display potential anti-aging, antioxidant, and organ-protective effects on the liver, brain, and kidney against D-gal-induced aging toxicity, slowing the aging process (163). The polysaccharide of *Hypsizygus ulmarius* containing galactose (44.24%), glucose (34.27%), mannose (15.61%) along with the small amount of xylose (3.33%), fucose (1.33%), and rhamnose (1.20%) has been shown to have a significant hepatoprotective effect against acute alcoholic liver damage in rats, as it protects the biological system from oxidative stress (26). *Ganoderma lucidum* polysaccharides with a molecular weight of 15.0 kDa have been found to reduce cognitive impairments in transgenic Alzheimer's disease (AD) mice by boosting neural progenitor cell proliferation (164). The polysaccharide-peptide complex of *Cordyceps militaris* consisting of mannose, glucose, and galactose with a molar ratio of 2.0:11.4:1 has been shown to regulate the lncRNA-miRNA-mRNA axis, thereby improving atherosclerosis (165). In conclusion, EMPs possess multiple bioactivities and play a positive role in maintaining bodily health.

## 5. Application prospect of EMPs

Due to their potential health benefits, EMPs can be utilized as additives in various food products, including bread, biscuits, and fruit juice, to enhance their nutritional value and health-promoting properties. For example, incorporating polysaccharides from *Lentinula edodes* into bread and rice muffins has been shown to improve their texture and nutritional quality (166, 167). Similarly, *Helvella leucopus* polysaccharides have been added to a compound beverage to enhance its antioxidant activity (168). In addition, the inclusion of 0.1–0.5% polysaccharides from *Lentinula edodes, Pleurotus eryngii*, and *Flammulina velutipes* in yogurt has been found to help probiotics survive, maintaining at least 107 CFU/ml at 4°C (169).

EMPs have also been utilized as dietary supplements to boost immunity and alleviate fatigue, with extracts of *Ganoderma lucidum* polysaccharides being a common ingredient (170). Furthermore, extracts of *Lentinus edodes* polysaccharides have been utilized in functional foods to aid in regulating blood glucose levels and preventing diabetes (171). Additionally, due to their antioxidant and anti-aging properties, EMPs have been utilized in cosmetics and personal care products (172). For example, *Tremella fuciformis* polysaccharides can protect skin cells against damage caused by ultraviolet (UV) radiation by decreasing the generation of reactive oxygen species and inhibiting the expression of matrix metalloproteinases. Moreover, this polysaccharides have demonstrated the potential to enhance skin hydration and elasticity, while diminishing the appearance of wrinkles triggered by UV exposure (173).

In addition, EMPs can be blended with other materials to create a composite material with excellent strength and toughness, which can be utilized in the production of food packaging materials. For example, blending mushroom polysaccharides with cellulose nanofibers to create a film can improve the film's tensile strength and elongation at break, while also decreasing its water vapor and oxygen permeability. As a result, the film exhibits superior moisture resistance and oxygen barrier properties (174).

Although EMPs have many potential health benefits and are widely used as a functional food ingredient, dietary supplement, pharmaceutical, and cosmetic products such as Immune Assist 24/7™, active hexose correlated compound (AHCC^®^), and MaitakeGold 404^®^, safety considerations should also be taken into account (175–177). The safety of EMPs depends on the source of mushrooms, the extraction method, and the purity of the polysaccharides. Some mushrooms may contain harmful compounds, such as heavy metals and mycotoxins, that can cause health problems if consumed in large amounts. Therefore, it is important to use mushrooms from reliable sources and to use safe extraction methods to obtain high-quality polysaccharides. In addition, the biological activity of EMPs are mainly revealed through *in vitro* and animal model experiments, and there is a lack of clinical trials to ensure that they do not cause harmful side effects in humans (178, 179). For example, the AHCC^®^ trial in patients resulted in mild gastrointestinal distress, headache, fatigue and foot cramps in 12% of the total patients (180). Therefore, further research is needed to explore the full potential of EMPs and to develop safe and effective applications in food and health products.

## 6. Conclusions and future perspectives

Through continuous investigation of the effects of EMPs on regulating the gut microbiome, mounting evidence suggests their potential to enhance human wellbeing. This review provides a comprehensive analysis of the methods used for extraction and purification of EMPs and the correlation between gut microbiome and disease. We also explore the interplay between EMPs and the gut microbiome, emphasizing their ability to preserve microbial equilibrium, bolster gut lining integrity, enhance short-chain fatty acid production, and modulate cellular signaling pathways for metabolic homeostasis and immune system reactivity. Despite the demonstrated benefits of EMPs as a natural form of treatment through regulation of the gut microbiome, there is still room for improvement in future studies.

With an emphasis on enhancing the structural analysis of EMPs and their relationship with the intestinal microbiota, there is a growing interest in investigating the impact of EMPs on gut health. The varying molecular mass and length of different EMPs play a crucial role in determining the recognition and digestion process of polysaccharides by gut bacteria. In recent years, there has been a growing emphasis on comprehending the structural-activity relationship of EMPs and their function in modulating the gut microbiota. However, there is still much to be understood regarding the relationship between the molecular weight, monosaccharide content, glycoside binding mechanism, and advanced structure of polysaccharides, and their effect on the gut microecology. Further validation is needed to clarify these relationships.

Conduct Further Clinical Trials for Safe and Effective Dosages of EMPs: Although both *in vitro* and *in vivo* animal studies have demonstrated the gut microbiota-modulating effects of EMPs, specific differences between humans and animals call for more clinical trials to be conducted. This will enable the development of tailored therapies that take into account the unique circumstances of individual patients and ensure safe and effective dosages of EMPs.

Conduct further research into the exact role of EMPs using omics technologies, and continue to investigate the specific degradation pathways of gut microbiota for EMPs, to provide a foundational understanding for personalized gut microbiota nutrition.

In summary, EMPs have rich nutritional value and medicinal value and have a wide application prospect in food, medicine, and cosmetics. Basic applied research on the healthcare value of EMPs should be further strengthened, as well as product development and standard setting in the fields of food, healthcare products, cosmetics, etc., to enhance human health or well-being.

## Author contributions

QLZ and YJ: conceptualization and writing original draft. QZ, HP, DLi, DLo, and XH: methodology. ZT and YZ: investigation and supervision. YZ: resources. LS, QZ, HP, DLi, DLo, and XH: writing—review and editing. All authors have read and agreed to the published version of the manuscript.
